# The novel compound STK405759 is a microtubule-targeting agent with potent and selective cytotoxicity against multiple myeloma *in vitro* and *in vivo*

**DOI:** 10.18632/oncotarget.11539

**Published:** 2016-08-23

**Authors:** Gabriela Rozic, Lena Paukov, Jana Jakubikova, Dikla Ben-Shushan, Adrian Duek, Adi Leiba, Abraham Avigdor, Arnon Nagler, Merav Leiba

**Affiliations:** ^1^ Division of Hematology and BMT, Sheba Medical Center, Tel Hashomer, Ramat Gan, Israel; ^2^ Sackler School of Medicine, Tel Aviv University, Tel Aviv, Israel; ^3^ Department of Medical Oncology, Jerome Lipper Multiple Myeloma Center, Harvard Medical School, Dana-Farber Cancer Institute, Boston, MA, USA; ^4^ Department of Medical Education, Mount Auburn Hospital, Harvard Medical School, Cambridge, MA, USA

**Keywords:** multiple myeloma, cell cycle, apoptosis, tubulin, AKT

## Abstract

Despite advances in treatment, multiple myeloma (MM) remains incurable. Here we propose the use of STK405759, a novel microtubule targeting agent (MTA) and member of the furan metotica family for MM therapy.

STK405759 inhibited tubulin polymerization in a cell-free system and in myeloma cells. This molecule had potent cytotoxic activity against several MM cell lines and patient-derived MM cells. Moreover, STK405759 demonstrated cytotoxicity against drug-resistant myeloma cells that overexpressed the P-glycoprotein drug-efflux pump. STK405759 was not cytotoxic to peripheral blood mononuclear cells, including activated B and T lymphocytes. This compound caused mitotic arrest and apoptosis of myeloma cells characterized by cleavage of poly (ADP-ribose) polymerase-1 and caspase-8, as well as decreased protein expression of mcl-1. The combination of STK405759 with bortezomib, lenalidomide or dexamethasone had synergistic cytotoxic activity. *In in vivo* studies, STK405759-treated mice had significantly decreased MM tumor burden and prolonged survival compared to vehicle treated- mice.

These results provide a rationale for further evaluation of STK405759 as monotherapy or part of combination therapy for treating patients with MM.

## INTRODUCTION

MM is characterized by clonal expansion of malignant plasma cells in the bone marrow leading to multiple bone lesions, anemia, renal injury and immunodeficiency [1]. MM represents 1% of all cancer diagnoses and comprises 2% of all cancer deaths. Autologous stem cell transplantation together with novel anti-MM agents such as thalidomide, lenalidomide (LEN), bortezomib (BTZ) and their analogues, has improved clinical outcomes. However, the development of drug resistance is universal and MM continues to be mostly an incurable disease [2–5]. Thus, the pursuit of newer alternative next generation therapeutic agents remains critically important.

MTAs function by affecting microtubules assembly/disassembly and have been shown to induce apoptosis in a wide variety of cancer cells, demonstrating a potent antitumor activity. The anti-mitotic drugs in current clinical use, including taxanes, epothilones and vinca alkaloids are all MTAs. However, the use of these compounds is limited primarily due to intrinsic or acquired resistance, usually correlated with overexpression of P-glycoprotein (Pgp) [6–8].

Recently, a novel set of MTAs named furan metotica has been described [9–10]. This group comprises a new chemotype that bind to tubulin in a unique manner. They prevent the outer kinetochore Ndc80 complex and the microtubule plus-end tracking protein CLIP-170 from binding to the taxol-stabilized microtubules. Until now, five members of this family have been shown to act as microtubule depolymerizing agents. Their advantages compared to other MTAs include their simple chemical structure, potent antitumor activity and ability to overcome drug-resistance.

This study evaluated the *in vitro* and *in vivo* anti-MM activity of STK405759, a novel member of the furan metotica family, and tested its potential as a MTA in preclinical models of human MM.

## RESULTS

### STK405759 reduces viability of MM cells

**Figure 1:**
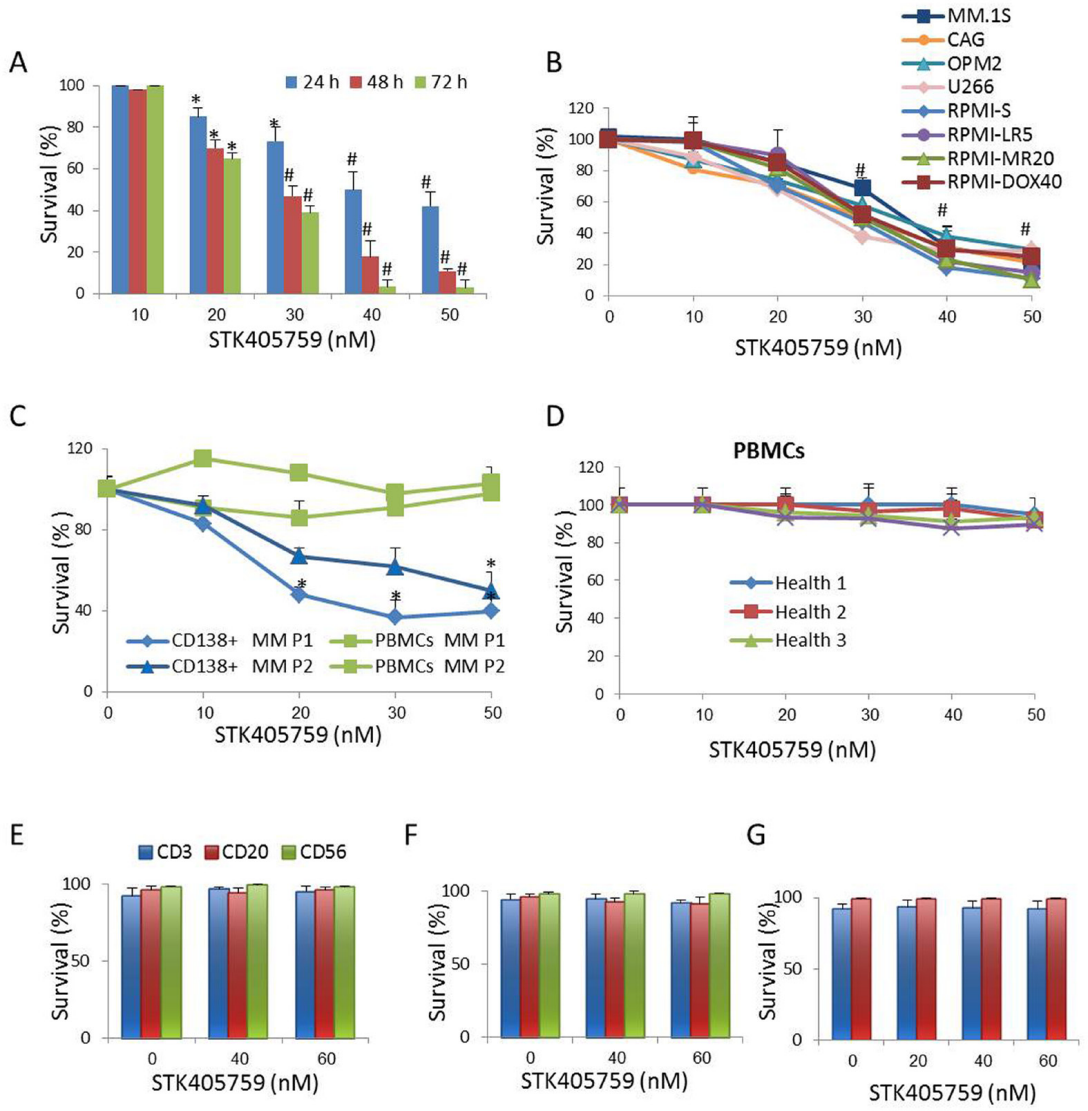
STK405759 induces cytotoxicity in MM cells Viability of cultured cells treated with different concentrations of STK405759 was assessed by XTT assay in **A.** RPMI-S MM cells exposed to the drug during 24, 48 and 72 h, **B.** a panel of MM cell lines, **C.** freshly isolated bone marrow CD138^+^ myeloma cells and PBMCs from MM patients and **D.** PBMCs from healthy donors exposure to STK405759 for 48 h. PBMCs isolated from **E.** healthy donors (n=5) and **F.** MM patients (n=5) were cultured and treated with STK405759 0, 40 and 60 nM during 48 h. The cells were resuspended in cell staining buffer and incubated with the appropriate antibody (CD3 for T cells; CD20 for B cells and CD56 for NK cells). Cell viability was analyzed by flow cytometry after adding PI for dead cell exclusion evaluation. **G.** B and T lymphocytes from healthy donors (n=3) were isolated and cultured in the presence of mitogens and STK405759 for 7 days. Cell viability was analyzed by flow cytometry. Each treatment was performed in triplicate in three independent experiments (cell lines) and presented as mean ± SE. Values were normalized to the drug-free control. Results are presented as mean ± SE. #p<0.05.

STK405759 significantly decreased survival of several human MM cell lines in a concentration- and time-dependent manner. Its cytotoxicity included MM cell lines resistant to anti-MM agents currently in use, such as RPMI-MR20 (mitoxantrone-resistant cells), RPMI-LR5 (melphalan (MEL)-resistant cells) and RPMI-DOX40 (doxorubicin (DOXO)-resistant cells) (Figure 1A-1B). The IC50 value after 48 h of treatment was less than 50 nM for each cell line tested. STK405759 also had selective cytotoxic activity on affinity-purified CD138+ MM cells from bone marrow samples of 2 MM patients. In contrast, peripheral blood mononuclear cells (PBMCs) from the same patients and from healthy donors were not affected by the cytotoxic effect of STK405759. In particular, there was no decrease in viability of cultured B lymphocytes, T lymphocytes and NK cells after 48h of STK405759 exposure (Figure 1E-1F). Similarly, the viability of T and B cells stimulated with mitogens did not decrease after 7 days of exposure to STK405759 at 20, 40 and 60 nM (Figure 1G).

**Figure 2:**
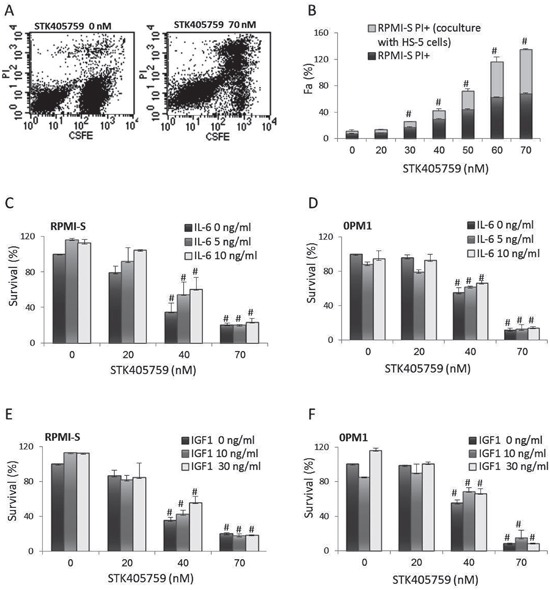
STK405759 overcomes the growth stimulatory effects of BMSCs, IL-6 and IGF1 on MM cells survival **A.** RPMI-S cells were stained with 5-(and 6)-carboxyfluorescein diacetate succinimidyl ester (CFSE), co-cultured with HS-5 and exposed to STK405759 (70 nM) for 48 h. The cells were counterstained with PI to distinguish live from nonviable cells using FACS analysis. **B.** The values of the fraction of nonviable MM cells cultured alone or with HS-5 stromal cells are presented as a function of STK405759 concentration. **C, E.** RPMI-S cells and **D, F.** OPM1 cells were cultured for 48 h at indicated STK405759concentrations in the presence of IL-6 (5 or 10 ng/ml) or IGF1 (10 or 30 ng/ml). Data presented are from three independent experiments and presented as mean ± SE. #p<0.05. Fa: fraction affected.

STK405759 caused cell death of RPMI-S cells co-cultured with HS-5 BMSCs (Figure 2A-2B). In contrast to Dexamethasone (DEX) [11–13], neither IL-6 nor IGF1 prevented STK405759 induced apoptosis of MM cells (Figure 2C-2F).

### STK405759 inhibits tubulin polymerization *in vitro* and decreases the insoluble fraction of microtubules mass in MM cells

**Figure 3:**
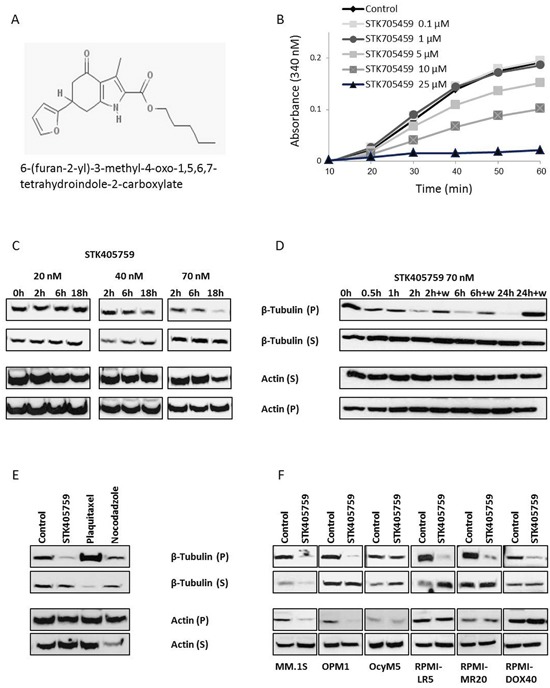
STK405759 exhibits a mechanism of action consistent with microtubules disruption **A.** Chemical structure of STK405759. **B.** The effect of STK405759 on microtubules polymerization was determined by a conventional turbidimetric assay. The assembly of bovine tubulin was monitored by an increase in absorbance at 340 nm. (C-F) The level of tubulin and actin in the polymerized (insoluble) and soluble protein fractions of MM cells were analyzed by immunoblotting in: **C.** RPMI-S cells treated with STK405759 20, 40 and 70 nM during the indicated times; **D.** RPMI-S cells treated with STK405759 70 nM during the indicated times. The cells were washed after 2, 6 and 24 h of treatment and the medium was replaced by fresh medium without STK405759 for additional 24 h; **E.** RPMI-S cells treated with STK405759 (70 nM), plaquitaxel (0.2 μg/ml) and nocodazole (100 nM) during 18 h and **F.** MM.1S, OPM1, OcyM5, RPMI-LR5, RPMI-MR20 and RPMI-DOX40 MM cells treated with STK405759 70 nM during 18 h. Blots are representative of two independent experiments. W: wash.

Given the similar chemical structure to that of STK405759 and the furan metotica MTAs family [9, 10], (Figure 3A), STK405759 was evaluated as a MTA. It inhibited the rate and extent of tubulin polymerization in a concentration-dependent manner in an *in vitro* cell-free system (Figure 3B) and in MM cells. We used Western blot to analyze the amounts of soluble and polymerized forms of tubulin in MM cells. STK405759 treatment of RPMI-S cells decreased their levels of polymerized tubulin respective to the free soluble tubulin fraction in a dose-dependent manner (Figure 3C) in as soon as 30 min and reached a maximum after 24 h of treatment (Figure 3D). This effect was reversible, since when RPMI-S cells were treated with STK405759, then washed and grown in medium without STK405759 for 24 h, the level of polymerized tubulin returned to that of untreated cells at the different time intervals tested (Figure 3D). As a control, treatment of MM cells with paclitaxel, an inhibitor of tubulin depolymerization, was associated with an increase in polymerized tubulin whereas treatment with nocodazole, a tubulin depolymerizing agent, was associated with a decrease in polymerized tubulin (Figure 3E).

Most of the tubulin protein was found in insoluble fractions in the different MM cell lines tested, including those resistant to mitoxantrone, MEL and DOXO. STK405759 treatment decreased the amount of polymerized tubulin in all cells tested (Figure 3F).

STK405759 also reduced the level of polymerized actin in RPMI-S, MM.1S and OPM1 cells after 18 h of treatment but had no effect on OcyM5 and MM cell lines resistant RPMI-LR5, RPMI-MR20 and RPMI-DOX40.

### STK405759 disrupts the morphology of mitotic spindles

**Figure 4:**
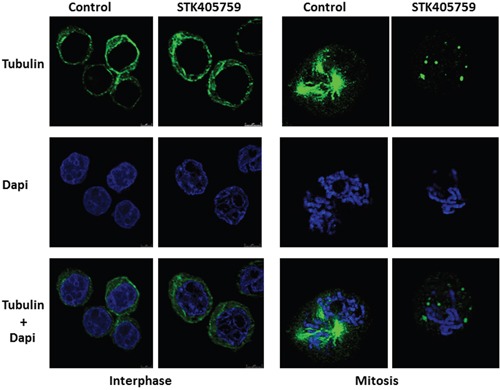
STK405759 altered spindle formation during mitosis RPMI-S cells were treated with 70 nM STK405759 for 12 h. The samples were fixed and stained with Alexa Fluor 488–conjugated antibody against β-tubulin (green). Counterstaining with DAPI for nuclear location and integrity was conducted during the sealing of the slides with antifade. Slides were examined using an inverted confocal microscope (Zeiss LSM780 Inverted Confocal Microscope – with multi-photon capabilities).

RPMI-S cells treated with 70 nM STK405759 for 12 h were fixed, stained for microtubules with anti-tubulin antibody and for chromatin with DAPI, and examined by confocal microscopy. There was no observable difference in the morphology of the microtubules during interphase between control and STK405759 treated cells. During mitosis, control RPMI-S cells presented a normal bipolar mitotic spindle with all chromosomes assembled in a compact central region between the two well separated spindle poles whereas STK405759 treated cells displayed abnormal chromosome and spindle microtubule organizations with the formation of multiple asters (Figure 4).

### STK405759 leads to cell cycle arrest and induces apoptosis

**Figure 5:**
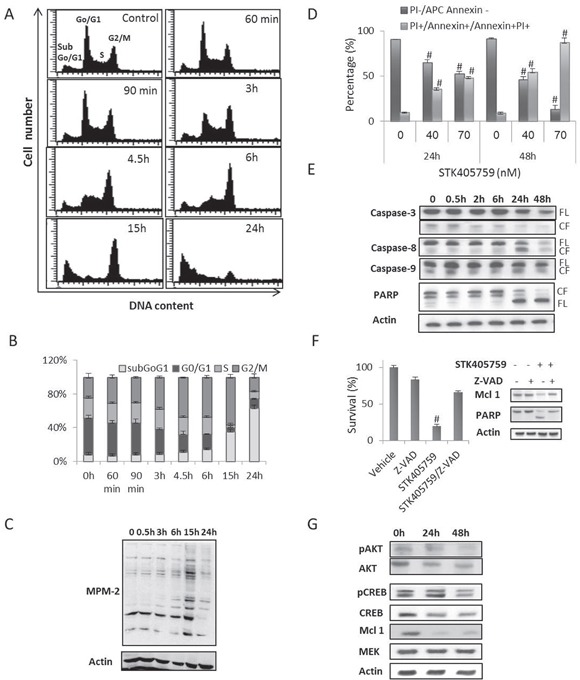
STK405759 inhibits proliferation, induces apoptosis and decreases AKT and CREB protein levels in MM cells **A, B.** RPMI-S cells were treated with STK405759 (70 nM) for different time intervals. Cell-cycle analysis showed an accumulation of cells in G2/M phase followed by an increase in apoptosis (subG0/G1). **C.** Lysates from RPMI-S cells treated with STK405759 70 nM during different time intervals and immunoblotted using anti MPM-2 antibody. **D.** RPMI-S cells were treated with STK405759 and analyzed for induction of apoptosis by Annexin V/PI assay. **E-G.** Lysates from RPMI-S cells treated with STK405759 70 nM during different intervals and immunoblotted using anti caspase-3, -8, -9, PARP and actin antibodies. FL, and CF indicate the full length and cleaved form, respectively and anti-pAKT, AKT, p-CREB, CREB, mcl-1, MEK and actin antibodies. (F) XTT assay of STK405759 treated cells in the presence of the apoptotic inhibitor Z-VAD-FMK (100 μM) during 24h. Blots are representative of three independent experiments. #p<0.05.

To determine whether STK405759 interrupted mitosis, the DNA content of MM cells was analyzed by flow cytometry after STK405759 treatment at various intervals within a 24 h period. STK405759 increased the percentage of RPMI-S cells in G_2_/M phase (Figures 5A-5B) as early as 3 h, and reached a maximal peak between 6 and 12 h of treatment. After 24 h of exposure, most of RPMI-S cells were at sub G0/G1 phase of the cell cycle, indicative of cell death.

To distinguish between distribution of G2 and M phases, cells were incubated with the MPM-2 antibody that detects proteins phosphorylated at the onset of mitosis [14]. Compared to control RPMI-S cells, STK405759 treated cells showed increased MPM-2 protein levels that reached a maximum at 15 h and then declined through 24 h of treatment (Figure 5C).

As mitotically arrested cells frequently undergo apoptosis, it was considered of interest to examine whether this was the cytotoxic mechanism of STK405759. STK405759 increased apoptosis of MM cells in a time- and concentration-dependent manner (Figure 5D). The induction of apoptosis was supported by the cleavage of caspase-8 and poly (ADP-ribose) polymerase (PARP) in MM treated cells (Figure 5E) and by the decreased in myeloid cell leukemia-1 (mcl-1) protein level. Treatment with the apoptotic inhibitor Z-VAD-FMK prevented STK405759 induced cell death and the changes induced by STK405759 in the expression of the apoptotic related proteins mcl-1 and PARP (Figure 5F-5G).

STK405759 reduced the expression of pAKT/AKT and pCREB/CREB in a time-dependent manner in MM treated cells without changing the level of MEK protein (Figure 5G).

### Sensitivity of MM cells to MTAs

**Figure 6:**
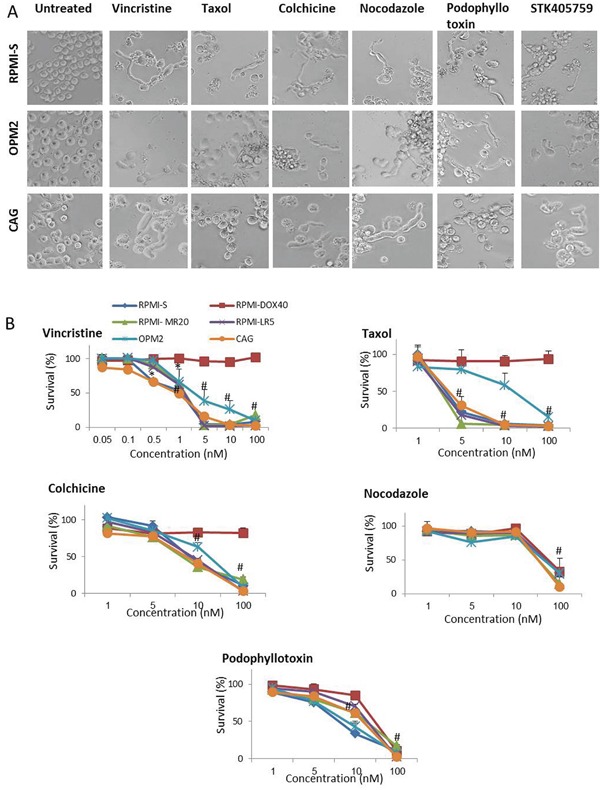
MTA change the morphology and induce cytotoxicity in MM cells **A.** Morphologic changes of RPMI-S, OPM2 and CAG MM cells treated with 100 nM vincristine, taxol, colchicine, nocodazole, podophyllotoxin and STK405759 during 18 h. **B.** Viability of MTA treated cells was assessed by XTT assay in RPMI-S, OPM2 and CAG MM cells treated with different concentrations of vincristine, taxol, colchicine, nocodazole, and podophyllotoxin for 48 h. Each treatment was performed in triplicate in three independent experiments and presented as mean ± SE. Photomicrographs were examined using an Olympus IX-70 microscope and images were processed using Olympus DP controller imaging software. #p<0.05.

The morphology of cells treated with STK405759 and other MTAs was evaluated by optical microscopy (Figure 6A). Each of the MTAs tested, including vincristine, taxol, colchicine, nocodazole, podophyllotoxin and STK405759 elongated the cytoplasm on MM cell lines (RPMI-S, OPM2 and CAG cells), that was visible after 12-18 h of treatment and then disappeared (Figure 6A).

All MTAs tested killed 50% of MM cells at nanomolar range concentration emphasizing the high responsiveness of MM cells (Figure 6B). RPMI-DOX40 cells which overexpress Pgp, [15, 16] remained fully sensitive to STK4057579 (Figure 1B), as did nocodazole and podophyllotoxin (Figure 6B) but were resistant to vincristine, taxol and colchicine treatment. Therefore, compare to other MTAs, STK405759 cause similar morphologic changes and potent cytotoxic activity on MM cells but overcame some forms of resistance.

### STK405759 enhances cytotoxicity of conventional and novel anti-MM therapies

The response of RPMI-S and MM.1S cells to STK405759 treatment in combination with BTZ, LEN, DEX, MEL and DOXO was evaluated by XTT assay (Table 1). The data obtained indicated that STK405759 triggered a synergistic effect when combined with LEN, DEX and BTZ (CI<1). The co-treatment of STK405759 with MEL or DOXO had an antagonist cytotoxic effect (CI >1) according to the Chou-Talalay method [17, 18].

**Table 1:** STK405759 combinatorial effect with standard anti-MM agents

		Combination Index	
		STK405759 45 (nM)	
Treatment		MM.1S	RPMI-S
**Bortezomib**	1 (nM)	0.62 ± 0.14	0.88 ± 0.02
	5 (nM)	0.58 ± 0.02	0.78 ± 0.02
**Lenalidomide**	5 (μM)	0.62 ± 0.14	0.77 ± 0.09
	25 (μM)	0.58 ± 0.02	0.74 ± 0.05
**Dexamethasone**	1 (nM)	0.76 ± 0.15	0.76 ± 0.06
	5 (nM)	0.63 ± 0.09	0.84 ± 0.03
**Melphalan**	2.5 (μM)	1.5 ± 0.10	1.08 ± 0.09
	5 (μM)	1.42 ± 0.06	1.14 ± 0.05
**Doxorubicin**	10 (nM)	1.30 ± 0.06	1.44 ± 0.01
	30 (nM)	1.54 ± 0.08	1.63 ± 0.10

RPMI-S and MM.1S cells were cultured for 48 h with STK405759 (45 nM), in combination with DEX (1 and 5 nM), LEN (5 and 25 μM), BTZ (1 and 5 nM), MEL (2.5 and 5 μM) and DOXO (10 and 30 nM). CI value <1, =1, >1 indicates synergism, additive and antagonist effect, respectively. Each treatment was performed in triplicate in three independent experiments and presented as mean ± SE).

### STK405759: *in vivo* anti-myeloma efficacy

**Figure 7:**
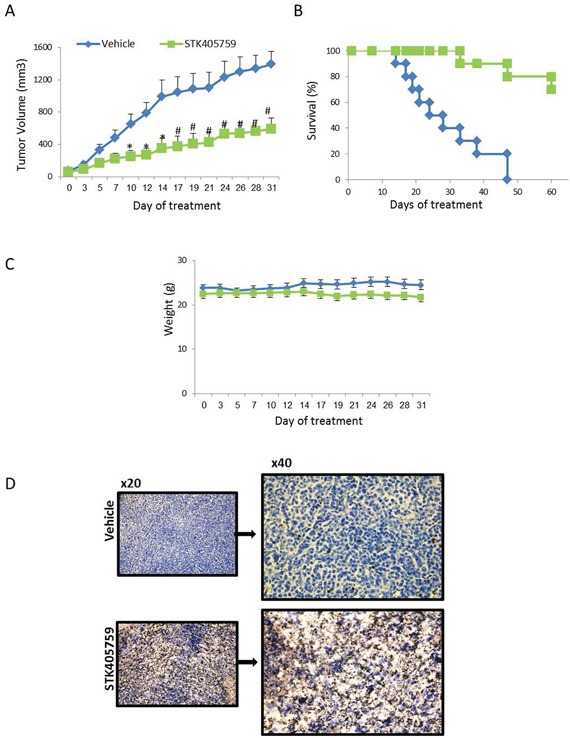
Low-dose STK405759 decreases tumor growth and improves overall survival in a MM xenograft mouse model SCID mice were given subcutaneous inoculations with 7 x10^6^ RPMI-S cells. Treatment was started when tumors reached 40-70 mm^3^ (vehicle or 0.5 mg/kg STK405759 once a day for 5 days a week, ip). **A.** Tumor burden was measured every 2-3 days using a caliper. Tumor volume is presented as mean ± SE. **B.** Kaplan-Meier curve and log-rank analysis of overall survival of STK405759 as compared with control treated mice. **C.** Body weight was evaluated 3 times a week. **D.** Representative microscopic images of tumor sections from control and STK405759-treated mice stained with TUNEL are shown. The slides were examined using an Olympus BX-43 microscope, and images were processed using cellSens entry digital imaging software. (Original magnification x20, x40). *p<0.005; # p<0.001.

STK405759 inhibited tumor growth (P<0.0005, t-test, Figure 7A) and increased the overall survival of treated mice (P = 0.0001, log rank, Figure 7B) as compared to control mice. No significant changes in weight or other signs of potential toxicity were observed during STK405759 treatment (Figure 7C). Sections from tumors of treated mice showed increased apoptosis compared to controls confirming the previously discussed *in vitro* results (Figure 7D).

## DISCUSSION

The current study showed that STK405759 is a novel MTA that induced MM cytotoxicity *in vitro* and *in vivo*. STK405759 was active at low concentrations against a broad range of MM cell lines and patient-derived MM cells, regardless of their sensitivity to conventional and novel therapies. This compound was not toxic towards PBMCs, including activated B and T cells. STK405759 treatment overcame MM cells resistance mediated by the presence of BMSCs. STK405759 showed synergistic, cytotoxic activity in MM cells when combined with BTZ, LEN or DEX. In an *in vivo* xenograft mice model of MM, STK405759 induced a significant reduction in tumor burden and prolonged overall survival. Taken together, these results show that STK405759 has potent and selective cytotoxic activity in preclinical models of MM.

STK405759 prevented tubulin polymerization in a cell-free system and in viable MM cells. MTAs have been suggested as potential MM drugs [19–26]. However, the use of plaquitaxel and docetaxel was shown to be ineffective in relapsing refractory MM [19, 21] and the effectiveness of thalidomide and vincristine was limited by toxicity and development of drug resistance [22, 28]. In MM, Pgp overexpression was associated with vincristine, DOXO, etoposide, glucocorticoid and carfilzomib resistance [16, 29–32]. We showed that RPMI-DOX40, the Pgp-positive multidrug resistant subline, remained fully sensitive to STK405759 cytotoxicity. In agreement with our results, other members of the furan metotica family have also been shown to evade Pgp activity in drug-resistant cancer cells [10]. Compare to flubendazole and nocodazole, MTAs that also overcame Pgp- drug resistance, STK405759 had the lowest IC50 value and required a lower dose to decrease tumor burden in mice [33, 34].

The disruption of microtubules dynamics caused by STK405759 treatment led to mitotic cell arrest as soon as 3 h after treatment and inactivation of AKT signaling (following 14 hours of treatment; data not shown). Cell death was triggered by activation of pro-apoptotic proteins (caspase-8 and PARP) and by decreased levels of the mcl-1 anti-apoptotic protein (following 24 hours of treatment).

Constitutive activation of the AKT kinase is considered a key oncogenic signal in MM and is associated with poor patient prognosis and drug resistance [35, 36]. STK405759 induced significant inhibition of AKT activity and expression in MM cells. Localization of AKT to microtubules sustains its activity, while disruption of microtubules attenuates AKT signaling independently of its initial activation [37]. Thus, the decreased AKT signaling observed following STK405759 treatment might be a consequence of tubulin destabilization.

Mcl-1 is over-expressed in cells from MM patients, acts as a survival protein, and correlates with relapse and short survival [38–40]. Accumulation of mcl-1 triggered by proteasome inhibition confers MM cell resistance to BTZ-induced lethality [41]. Thus, STK405759 treatment, may play a role as a single agent against myeloma cells that depend on decreased mcl-1 for survival, and in combination with other drugs where mcl-1 overexpression is the main mechanism of resistance.

In summary, the advantages of STK405759 as a MTA include the simplicity of its chemical structure which may help prevent acute toxicity, its potent ability to overcome drug resistance and its ability to decrease AKT and mcl-1 expression.

Taken together, the present study demonstrated that STK405759 is a novel MTA with promising anti-MM activity and provided a framework for its use, alone and in combination with current therapies.

## MATERIALS AND METHODS

### Compounds

STK405759 was synthesized and provided by Vitas-M Laboratory (Apeldoorn, Netherlands). MEL, DEX, DOXO, vincristine, colchicine, taxol, nocodazole and podophyllotoxin were purchased from Sigma-Aldrich (St. Louis, MO, USA). LEN and BTZ were provided by Selleckchem (Houston, TX, USA). Z-VAD–FMK was purchased from Apexbio Technology, Houston, TX, USA.

### Cell lines

Human MM cell lines RPMI 8226 (RPMI-S), MM.1S, U266 and the human BM stromal cell (BMSCs) line HS-5 were purchased from ATCC, Manassas, VA, USA. The RPMI sublines RPMI-MR20, RPMI-LR5 and RPMI-DOX40 and the CAG, OPM1 and OPM2 cell lines were kindly provided by Jana Jakubikova (Dana-Farber Cancer Institute, Boston, MA, USA). MM cell lines were grown in RPMI-1640 medium and HS-5 in Dulbecco's modified Eagle medium (Gibco/BRL, Gaithersburg, MD, USA), both supplemented with 10% fetal calf serum and antibiotics (Biological Industries, Beit Haemek, Israel).

### Cell viability assay

MM cell lines were plated at 1-2 x10^4^ cells per 96-well and treated with different concentrations of STK405759. For patient samples, bone marrow aspirates were collected after obtaining signed informed consent in accordance with regulations of Chaim Sheba Medical Center, Tel Aviv, Israel. Bone marrow and peripheral blood were processed by lymphoprep (Axis-Shield PoC, Oslo, Norway) to isolate mononuclear cells and myeloma cells were purified using CD138 microbeads (Miltenyi Biotec, Bergisch Gladbach, Germany). Cell viability was measured using XTT cell proliferation Kit (Biological Industries, Beit Haemek, Israel) according to manufacturer's instructions. Recombinant human interleukin 6 (IL-6) and insulin-like growth factor 1 (IGF1) (PeproTech, Rocky Hill, NJ, USA) were used in cultures of RPMI-S cells, as indicated.

### PBMCs subpopulations viability assay

Peripheral blood samples from 5 healthy donors and 5 MM patients were processed by lymphoprep to isolate PBMCs. The isolated cells were plated at 2×10^4^ cells per 96 well and exposed to different concentrations of STK405759 for 48h. The cells were resuspended in Cell Staining Buffer, incubated with CD3, CD20 and CD56 antibodies (Becton Dickinson Biosciences, San Jose, CA, USA) and stained with PI to distinguish live from non-viable cells. Data were collected using FACS Calibur and analyzed with CellQuest software (Becton Dickinson Biosciences, San Jose, CA, USA).

### B and T lymphocytes stimulation

PBMC were isolated from heparinized venous blood by Ficoll gradient centrifugation. B cells were isolated with anti-CD19 microbeads (Miltenyi Biotec, Bergisch Gladbach, Germany). B and T cells were cultured (2.0 × 10 ^6^ cell/ml) with RPMI-1640 medium containing 10% fetal calf serum, L-glutamine, penicillin/streptomycin (Gibco/BRL, Gaithersburg, MD, USA), 1% HEPES buffer and β-mercaptoethanol (Sigma-Aldrich, St. Louis, MO, USA). Purified B lymphocytes cultures were stimulated with pokeweed mitogen (PWM, 10 μg/mL), PMA (12-O-tetradecanoylphorbol 13-acetate; 10 ng/mL) and calcium ionophore (1 μg/mL). T cells were stimulated with IL-2 (10 ng/ml, PeproTech, Rocky Hill, NJ, USA), PMA (10 ng/mL) and ionomycin (1 μg/mL; all Sigma-Aldrich, St Louis, MO, USA). The cells were treated with STK405759 20, 40 and 60 nM during 7 days. The medium and reagents were replenished every 48 h. Viability of the cells was quantitated by flow cytometry staining with fluorochrome-conjugated monoclonal antibodies (aCD20 for B cells and aCD3 for T cells) and PI.

### Co-culture experiments

RPMI-S cells, previously stained with CFSE (Thermo Fisher Scientific, Inc., Waltham, MA, USA), were added to wells seeded with HS-5 BMSCs and exposed to STK405759 treatment for 48 h. Then, the cells were collected and stained with PI. Data were collected using FACS Calibur and analyzed with CellQuest software (Becton Dickinson Biosciences, San Jose, CA, USA).

### Cell-free tubulin polymerization assay

This assay was performed following manufacturer's instructions (Cytoskeleton, Denver, CO). The reaction was conducted in the presence of 15% glycerol and 3 mg/ml tubulin. Drugs were dissolved in DMSO and added to the reaction mixtures; the final concentration of DMSO was <2%. Tubulin polymerization was monitored by measuring OD340 at 37°C in a Synergy 4 microplate reader (BioTek Instruments, Winooski, USA).

### Analysis of microtubules polymerization in MM treated cells

RPMI-S cells were exposed to STK405759 for varying intervals. Cells were lysed in microtubules stabilizing buffer (20 mM Tris–HCl, pH 6.8, 0.14M NaCl, 1 mM EGTA, 0.5% NP-40, 1 mM MgCl2, 0.4 μg/ml paclitaxel, protease inhibitor mixture (Complete; Roche Diagnostics), protease inhibitor cocktail 1 and 3 (Sigma-Aldrich, St. Louis, MO, USA) and 1 mM phenylmethylsulfonyl fluoride) and centrifuged at 12,000 rpm for 10 min. The supernatants containing soluble tubulin and the pellets containing polymerized tubulin were collected and subjected to immunoblot analysis with anti-tubulin (Sigma-Aldrich, St. Louis, MO, USA) and anti-actin (Santa Cruz Biotechnology, CA, USA) antibodies.

### Immunofluorescence staining

RPMI-S cells were exposed to 70 nM STK405759 for 12 h and fixed in PBS containing 4% formaldehyde for 15 minutes, washed in PBS and permeabilized in 0.3% Triton X-100/PBS (10 minutes at 37°C and 10 minutes at 4°C). After blocking with 3% bovine serum albumin in PBS, the samples were incubated with Alexa Fluor 488–conjugated antibody against β-tubulin overnight at 4°C. While sealing the slides with antifade, they were counterstained with DAPI for nuclear location and integrity. Slides were examined using an inverted confocal microscope (Zeiss LSM780 Inverted Confocal Microscope).

### Cell cycle analysis

RPMI-S cells were exposed to 70 nM STK405759 for varying intervals, permeabilized by 70% ethanol at −20°C overnight and incubated with 50 μg/ml PI (Becton Dickinson Biosciences, San Jose, CA, USA) and 20 units/ml RNase-A (Roche Diagnostics, Mannheim, Germany). DNA content was analyzed by flow cytometry. Data were collected using FACS Calibur and analyzed with CellQuest software (Becton Dickinson Biosciences, San Jose, CA, USA).

### Flow cytometry analysis of apoptosis

RPMI-S cells were treated with 40 and 70 nM STK405759 for 0, 24 and 48 h. For evaluation of apoptosis, cells were processed using an Annexin V/PI kit (Becton Dickinson Biosciences, San Jose, CA, USA) according to manufacturer's instructions. Data were collected using FACS Calibur and analyzed with CellQuest software (Becton Dickinson Biosciences, San Jose, CA, USA).

### Immunoblotting analysis

For immunoblotting analyses, MM cell lines were plated in RPMI 1640 medium with 10% FBS and antibiotics. STK405759 (70 nM) was added for various time intervals. Cells were lysed in RIPA lysis buffer containing 10 mM sodium pyrophosphate, 2 mM sodium orthovanadate, 5 mM sodium fluoride, 5 g/ml aprotinin, 5 g/ml leupeptin, and 1 mM phenylmethylsulfonyl fluoride (Sigma-Aldrich, St. Louis, MO, USA). Proteins were separated by sodium dodecyl sulfate-polyacrylamide gel electrophoresis, transferred onto nitrocellulose membranes and immunoblotted with anti- caspase-3, caspase-8, caspase-9, PARP (Cell Signaling Technology, Beverly, MA, USA), mcl-1 phospho AKT (Ser473), AKT, MEK, gapdh and actin antibodies (Santa Cruz Biotechnology, CA, USA) and phospho Ser/Thr-Pro mitotic protein (MPM-2) antibody (Millipore). Immunoreactive bands were detected by Western Blot chemiluminescence reagents (Thermo Fisher Scientific Inc, Waltham, MA USA) and exposed on X-Ray film (Fujifilm Corporation, Tokyo, Japan).

### Xenograft murine model

Male CB-17/IcrHsd-Prkdc-scid mice (6-8 weeks old) were maintained in accordance with Institutional Animal Care Use Committee guidelines. Mice were housed in the Animal Research Facility of Chaim Sheba Medical Center and experiments were performed in accordance with approved protocols. During the experiment, the mice were gamma-irradiated (150 rads) using Cs137 γ-irradiator source and 24 h post-irradiation, injected subcutaneously with 7 x10^6^ RPMI-S cells suspended in PBS. Two weeks later, when tumors reached 40-70 mm^3^, mice were randomized into two groups (10 mice/group), and the following treatment protocol was implemented. Group 1: control (DMSO) administered intraperitoneally (ip) 5 days a week throughout the duration of experiment. Group 2: STK405759 (0.5 mg/kg (administered ip 5 days a week throughout the duration of experiment. Changes in body weight and tumor burden were evaluated every 2 to 3 days. Tumor volumes were measured by a caliper every other day and calculated using the following formula: length x width^2^ x 0.5. Mice were sacrificed in accordance with institutional guidelines when tumors reached 1.5 cm^3^ or if the mice appeared moribund, to prevent unnecessary morbidity to the mice. Tumors were immediately collected from the mice and analyzed by histochemistry.

### Histochemistry

The tumors were fixed in 4% paraformaldehyde for 24 h (Sigma-Aldrich, St. Louis, MO, USA), washed with PBS, dehydrated in increasing alcohol concentrations and embedded in paraffin blocks. Sections were deparaffinized and rehydrated, treated with proteinase K (20 μg/ml) for 15 min and washed in PBS. Endogenous peroxidase was blocked with 3% hydrogen peroxide for 15 min. Fragmented nuclear DNA associated with apoptosis in histological sections was labeled *in situ* with digoxigenin-deoxyuridine (dUTP), introduced by terminal deoxynucleotidyl transferase (TdT), using ApopTag® peroxidase *in situ* apoptosis detection kit according to manufacturer's instructions (Intergen, Oxford, England, UK). The reaction was terminated using the ApopTag® stop buffer followed by anti-digoxigenin-peroxidase application and the labeled nuclei were detected with ACE substrate as the chromogen (Sigma-Aldrich, St. Louis, MO, USA). The slides were examined using an Olympus BX-43 microscope, and images were processed using cellSens entry digital imaging software.

### Statistical analysis

The differences in drug-treated vs. control cultures was determined using Student's t-test. Data are presented as mean ± standard error (SE). The IC50 value of each drug and the combinatorial index (CI) were calculated using CalcuSyn software [17, 18]. For *in vivo* experiments, survival was assessed using Kaplan-Meier curves and log-rank analysis.
